# The 2018 Bioinformatics Open Source Conference (GCCBOSC 2018)

**DOI:** 10.12688/f1000research.15936.1

**Published:** 2018-08-17

**Authors:** Nomi L. Harris, Heather Wiencko, Brad Chapman, Peter J.A. Cock, Karsten Hokamp, Hilmar Lapp, Chris Fields, Bastian Greshake Tzovaras

**Affiliations:** 1 Lawrence Berkeley National Laboratory, Berkeley, CA, 94707-2623, USA; 2 Open Bioinformatics Foundation, Dublin, Ireland; 3 Bioinformatics Core, Harvard T.H. Chan School of Public Health, Boston, MA, 02115, USA; 4 The James Hutton Institute, Dundee, DD2 5DA, UK; 5 Smurfit Institute of Genetics, Trinity College Dublin, Dublin, 2, Ireland; 6 Center for Genomic and Computational Biology, Duke University, Durham, NC, 27708, USA; 7 High Performance Computing in Biology Group, Carver Biotechnology Center, University of Illinois Urbana-Champaign, Urbana, IL, 61801, USA; 8 Open Humans Foundation, Sanford, NC, 27330, USA

**Keywords:** conferences, bioinformatics, open source, open science

## Abstract

In 2018, the annual Bioinformatics Open Source Conference was held for the first time in conjunction with the Galaxy Community Conference, as an experiment to see if we could reach people in the bioinformatics community who aren’t part of the audience attracted by ISMB. Held in June 2018 at Reed College in Portland, Oregon, GCCBOSC (Galaxy Community Conference and Bioinformatics Open Source Conference) attracted over 300 participants from around the world. The meeting started with two days of training, followed by two days of talks and poster/demo sessions (with some joint and some parallel sessions). The joint sessions included well-received keynote talks by Tracy Teal, Fernando Pérez and Lucia Peixoto, as well as a panel discussion about documentation and training. After the main meeting, many attendees stayed for up to four additional collaboration days, an extended version of the Codefests that have been held in conjunction with previous BOSCs. GCCBOSC was a successful experiment. The organizers concluded that the best way to serve the broadest community of potential BOSC attendees will be to partner some years with the International Society for Computational Biology (ISMB) and others with GCC.

## Introduction

**Figure 1.**
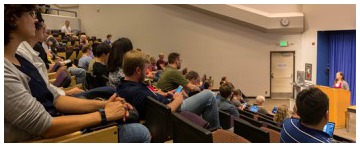
The audience at the Galaxy Community Conference and Bioinformatics Open Source Conference (GCCBOSC) listens to opening remarks by BOSC chair Nomi Harris. (All photographs in this article, unless otherwise credited, are from Bérénice Batut’s Flickr album, under a CC-BY-SA license.)

Together, the Galaxy Community Conference (GCC) and Bioinformatics Open Source Conference (BOSC) comprised the first Bioinformatics Community Conference. At the Galaxy Community Conference and Bioinformatics Open Source Conference (GCCBOSC 2018), participants were able to meet and collaborate with a broad community of bioinformatics developers and users who focus on open, interoperable software tools and libraries that facilitate scientific research ( Figure 1).

**Figure 2.**
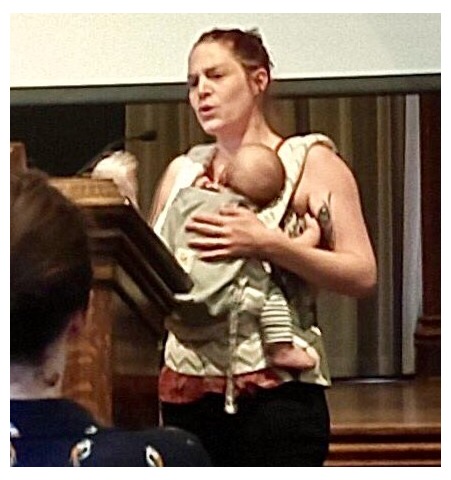
A Galaxy Community Conference and Bioinformatics Open Source Conference (GCCBOSC) speaker delivers her talk with her baby strapped to her. Photo by Allison Creason. Reproduced with permission from the photographer and the subject.

Every year from 2000 through 2017, BOSC was part of the ISMB conference. Partnering with GCC in 2018 was an experiment to see if we could reach people in the bioinformatics community who aren’t part of the audience attracted by ISMB. We had observed that GCC, and the Galaxy project in general, had successfully established a strong and growing community of participants. GCC includes training days, which we had not previously been able to offer as part of BOSC. Moreover, because GCC is a smaller conference than ISMB and is typically held at less expensive venues such as college campuses, the registration fees for GCCBOSC 2018 were very affordable. This was particularly welcome in the context of our ongoing attempts to lower the obstacles that make it hard for some would-be participants to attend. Additionally, thanks to sponsor funding we were able this year to offer subsidized child care and an onsite lactation room that enabled a speaker who would otherwise have been unable to attend to bring her four-month-old baby and participate actively in the meeting ( Figure 2).

## Training at GCCBOSC

**Figure 3.**
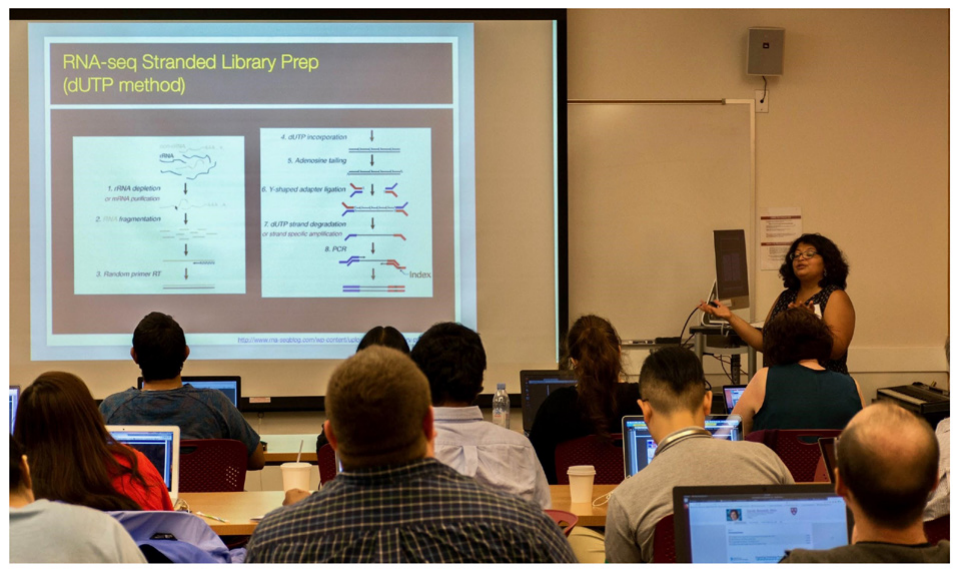
One of the many training workshops at the Galaxy Community Conference and Bioinformatics Open Source Conference (GCCBOSC).

GCCBOSC started with two days of parallel training sessions ( Figure 3) covering a range of bioinformatics topics. 199 people attended at least one of these, which ranged from an all-day Data Carpentry genomics workshop on “Data Organization and Automation” to short workshops on tools and techniques including RNA-seq, InterMine, Common Workflow Language, and many more. Post-meeting feedback from participants in the training workshops, who ranged from total beginners to expert developers, was very positive.

## Keynote talks

Like past BOSCs (and GCCs), GCCBOSC featured keynote talks from several recognized leaders in the field. Two of the keynote speakers were invited by BOSC, and the third was invited by GCC. The first BOSC keynote speaker was **Tracy Teal**, the Executive Director of The Carpentries. Her talk, “ Democratizing data,” started with an interactive exercise that got the whole audience thinking about what’s good and bad about the current state of bioinformatics, and how it can be improved. Tracy made the point that bioinformatics research is limited less by the data and tools than by who does the work, and how. What can be done to support the people who work in the field, and help them acquire the skills they need? The Software and Data Carpentry workshops aim to "democratize" data skills by reaching a diverse set of learners across the globe and teaching them core skills in data preparation and analysis, software development and communication.

BOSC keynote speaker **Fernando Pérez**, the co-founder of Project Jupyter, is a hero in the world of open and reproducible science. Jupyter Notebooks are widely used by biologists, physicists, social scientists, and pretty much anyone who wants to work with numbers. There are already well over 2 million Jupyter Notebooks on GitHub. Fernando’s talk focused on open source lessons from the Jupyter project. Fernando noted that he started working in open source for four reasons: ethical (openness is fair); human/social (openness fosters collaboration); epistemological (proprietary science is an oxymoron); and technical (Fernando invented IPython notebooks, the predecessor to Jupyter, as an easy and fun way to quickly analyze data using Python; making it open allowed others to extend it, including adding support for dozens of other programming languages). The Jupyter project has attained stunning success in developing a popular, highly extended tool and ecosystem as well as a vibrant, growing community, Fernando’s descriptions of how the project is run and how the community is nurtured were therefore of great interest to GCCBOSC attendees. Fernando closed by noting some of the barriers to inclusion and sustainability in the open source world, including the observation that when people are expected to work on open source projects for free, only the people who can afford to work for free can participate.

The closing keynote talk was delivered by **Lucia Peixoto**, who was invited by GCC. A computational biologist who studies brain function, Lucia--who thanked the conference for providing the subsidized child care that enabled her to attend--discussed how confounders can hinder reproducible science.

## Talks, posters, panel

**Figure 4.**
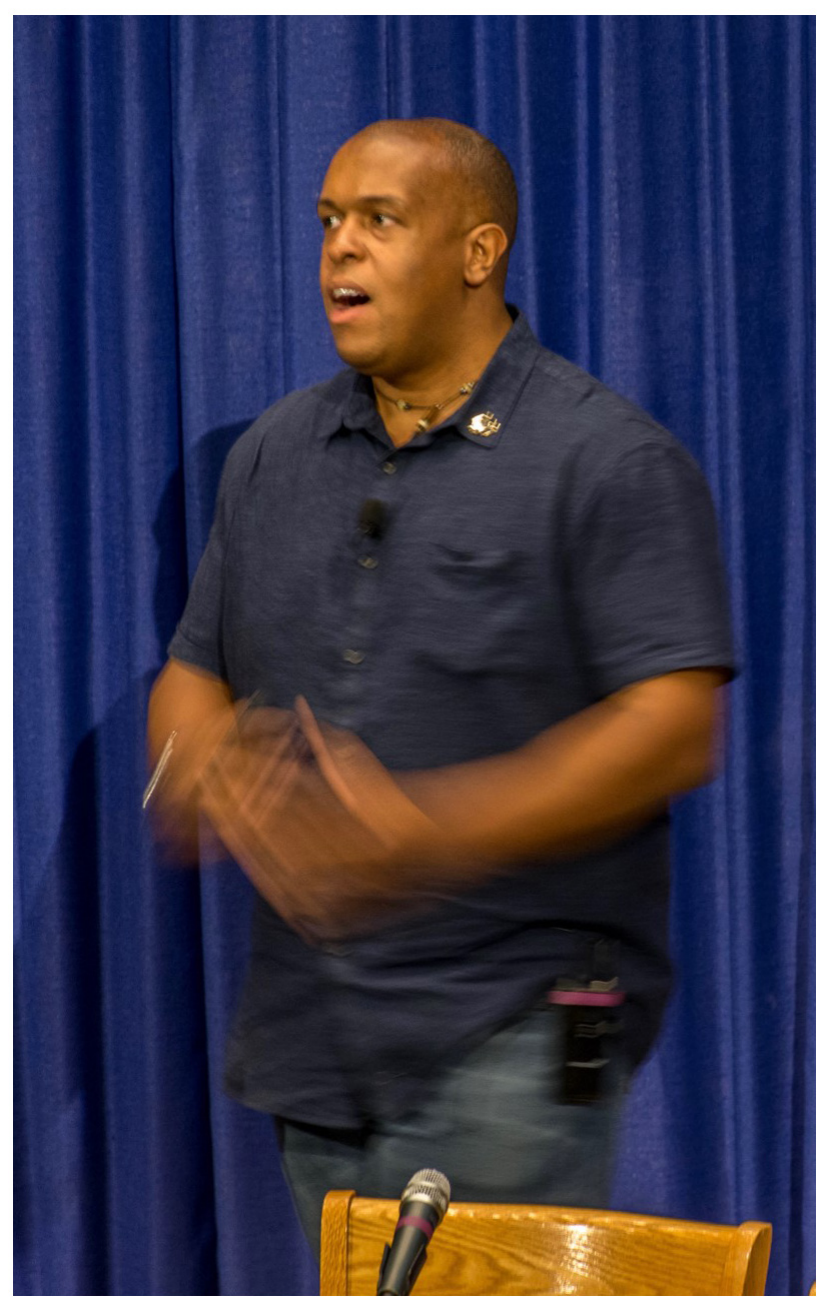
Jason Williams talks with his hands at the Galaxy Community Conference and Bioinformatics Open Source Conference (GCCBOSC).

GCCBOSC included two full days of talks, with joint sessions for the keynote talks and panel and parallel GCC/BOSC sessions for the talks chosen from submitted abstracts ( Figure 4). (The parallel sessions were one of the few aspects of the conference that some attendees found disappointing--they wanted to be able to attend the GCC talks ‘and’ the BOSC talks, but were forced to choose between them.) BOSC sessions this year were:

- Translational & Medical Informatics
- All About Data
- Developer Tools and Libraries
- Workflows
- Project Updates and Late-Breaking Research

Organizing committee member Brad Chapman put together two blog posts that give a good overview of the talks.

**Figure 5.**
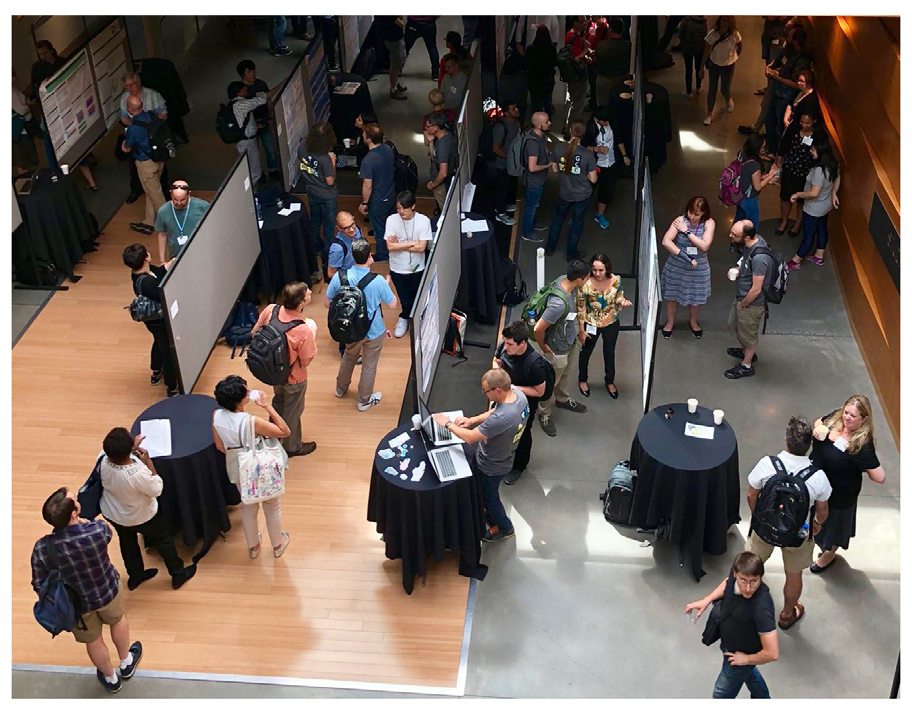
Galaxy Community Conference and Bioinformatics Open Source Conference (GCCBOSC) participants mingle at the poster/demo session.

**Figure 6.**
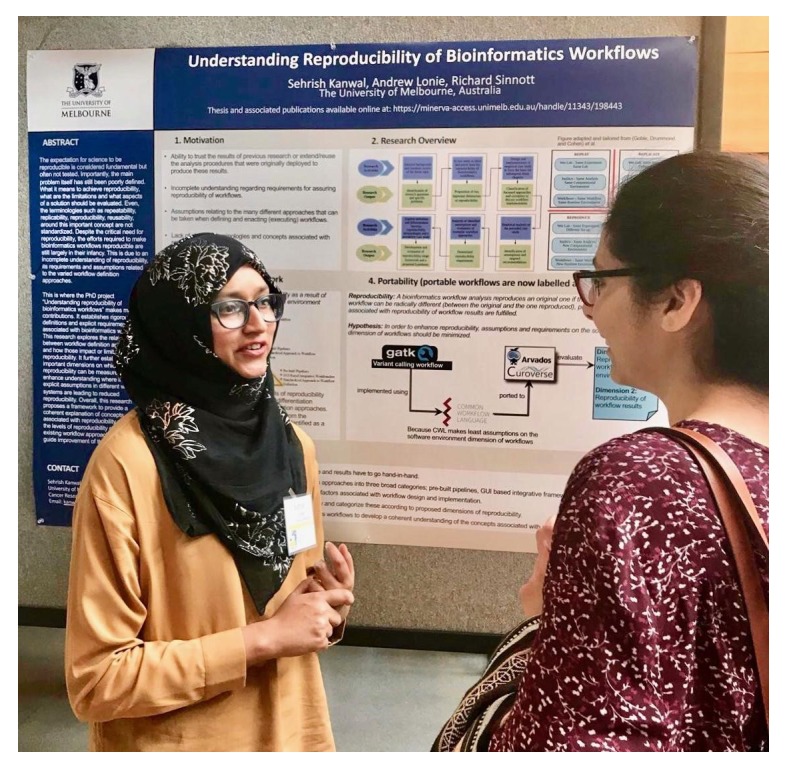
A poster presenter discusses her work with an attendee.

The well-attended poster/demo sessions at GCCBOSC included a total of 98 posters and demos. Tables were provided in the poster area for demonstrators’ laptops, something that had not been financially viable at previous BOSCs ( Figure 5 and Figure 6).

**Figure 7.**
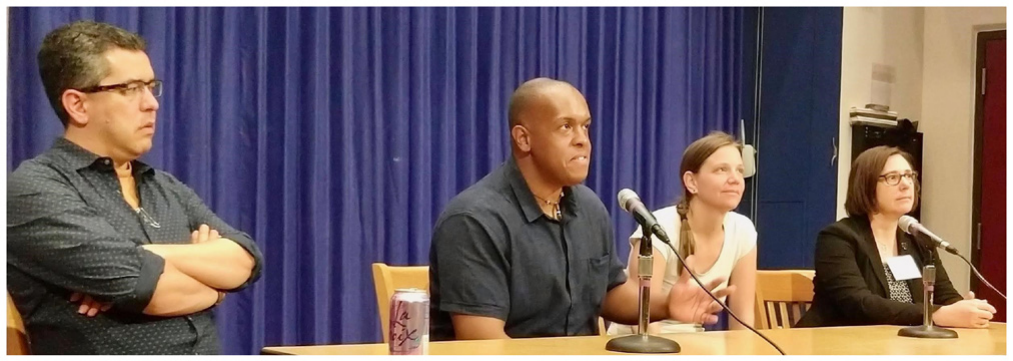
The four Galaxy Community Conference and Bioinformatics Open Source Conference (GCCBOSC) panelists (Fernando Peréz, Jason Williams, Bérénice Batut and Tracy Teal). Photo by Martin Čech, reproduced with permission under a CC-BY-SA license.

GCC, which had not previously included panels, was happy to participate in a joint panel session with BOSC ( Figure 7). The panel topic was training and documentation in bioinformatics--important but often overlooked aspects of every project. Panelists Bérénice Batut, Fernando Peréz, Tracy Teal and Jason Williams, with moderator Brad Chapman, discussed ways to lower the perceived stigma of working on documentation, improve its quality, and enable more users to understand, use and build on open software.

## Birds of a Feather

**Figure 8.**
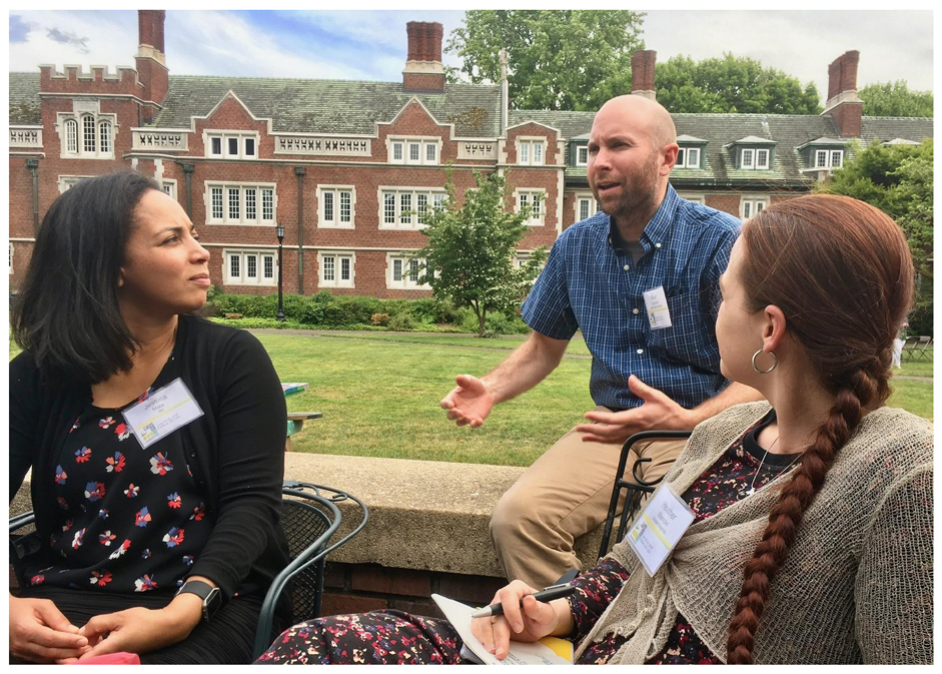
Participants in the Open Bioinformatics Foundation (OBF) Birds of a Feather at the Galaxy Community Conference and Bioinformatics Open Source Conference (GCCBOSC).

**Figure 9.**
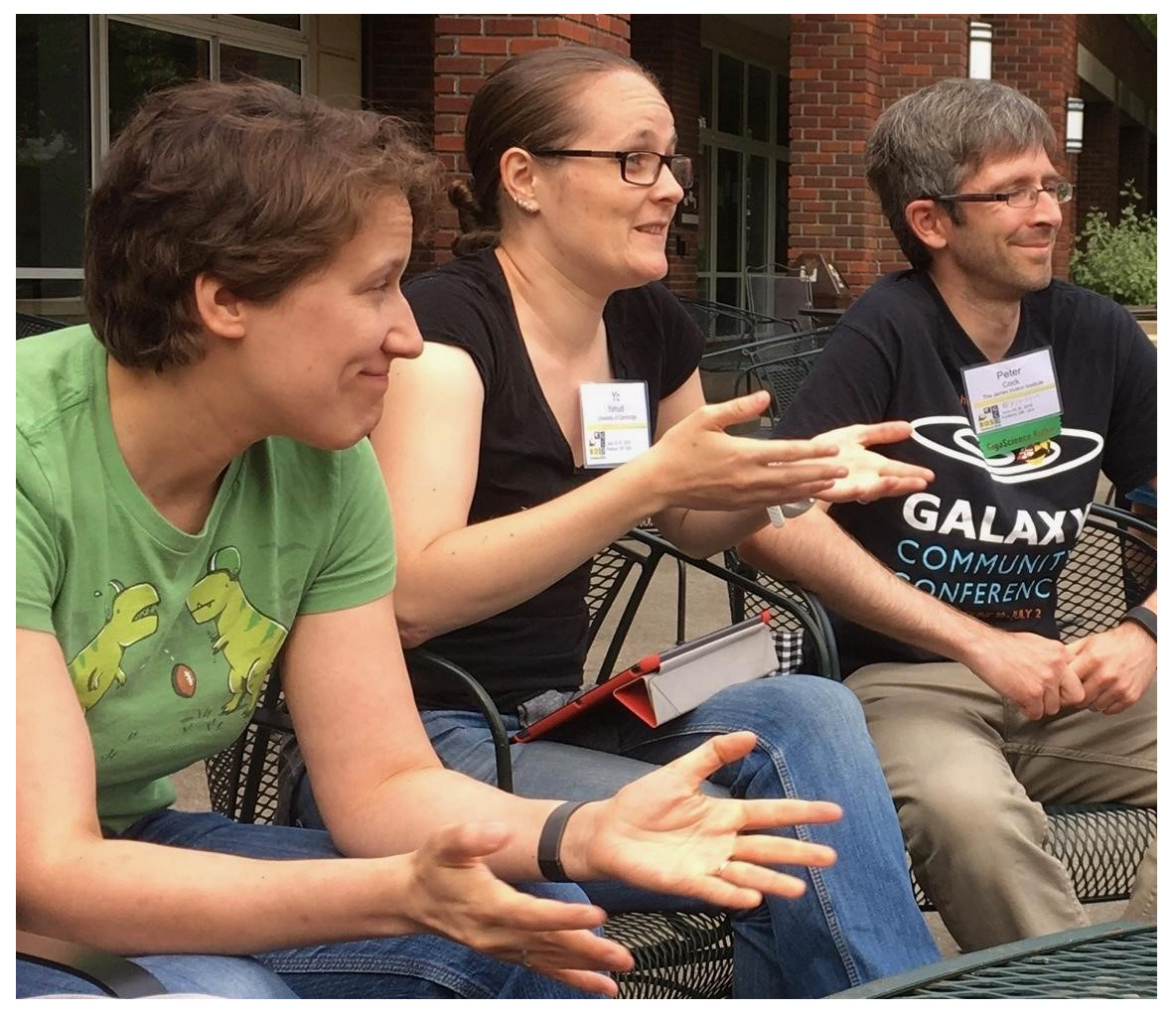
Participants in the Open Bioinformatics Foundation (OBF) Birds of a Feather at the Galaxy Community Conference and Bioinformatics Open Source Conference (GCCBOSC).

Birds of a Feather (BoFs), which are informal, self-organized discussion groups, are always a popular part of BOSC (and of GCC) ( Figure 8 and Figure 9). BoFs can help attendees learn more about new projects and communities, find collaborators, and interactively share feedback with project organizers. GCCBOSC 2018 had 18 BoFs, including:

- Getting contributors to your open project
- Stable and persistent URIs in biological data integration systems
- Educating Funders/PIs on Open Science
- Open Bioinformatics Foundation community (OBF)
- Common Workflow Language community chat
- Preparing for the next steps in AI

Other BoFs focused on topics such as Galaxy administration, Bioconda, and many more. Overall, about 75% of survey respondents who attended GCCBOSC said that they participated in one or more BoF.

## CollaborationFest

**Figure 10.**
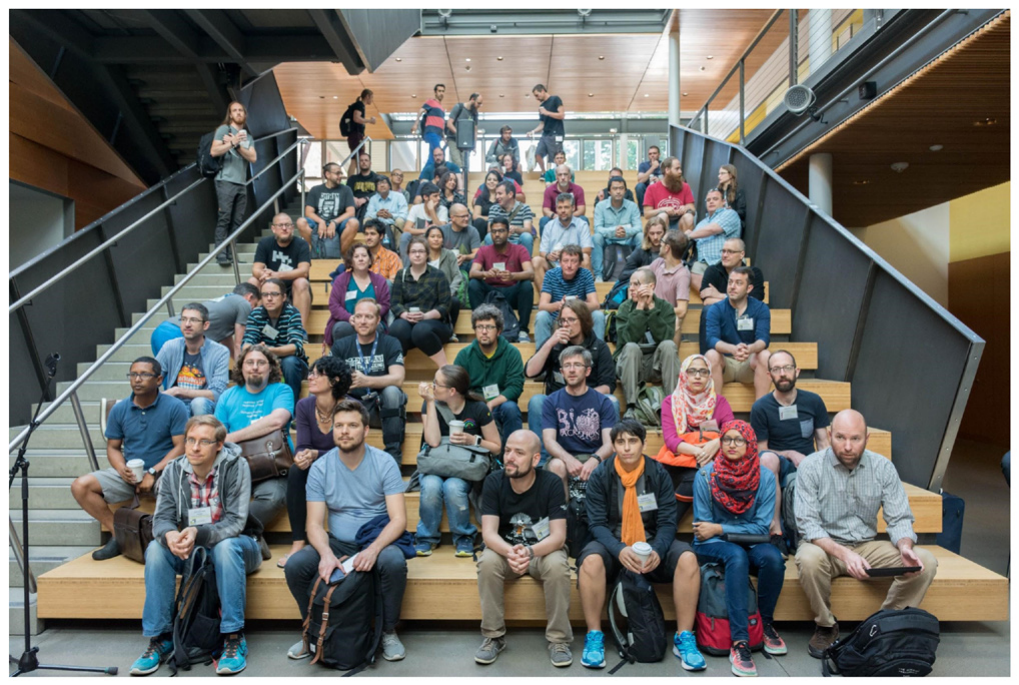
CoFest attendees assembled for morning meetings before breaking into smaller groups to work on collaborative projects.

**Figure 11.**
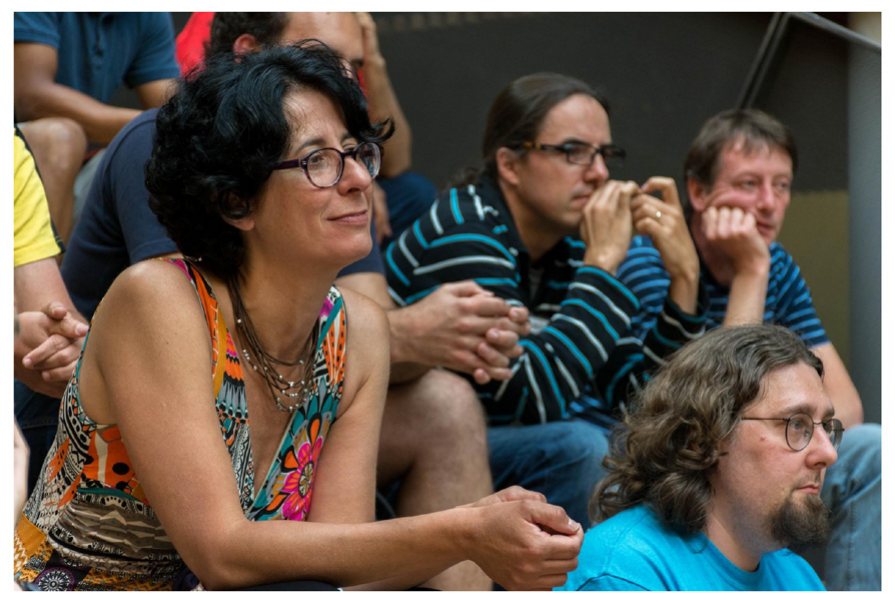
CoFest attendees assembled for morning meetings before breaking into smaller groups to work on collaborative projects.

GCCBOSC finished with an inclusive two-day CollaborationFest (or CoFest) event ( Figure 10, Figure 11), with an additional optional two-day CoFest Encore. The goal of the event is to build an active open bioinformatics community focused around solving the biological problems discussed at the training days and conference. Community members worked together on coding and debugging, building training materials, improving documentation and helping integrate new attendees. 160 people participated in the two-day CoFest (including a satellite Data Commons collaboration event)--nearly three times as many as previous CodeFests. Highlights of the CoFest ranged across multiple projects and included: onboarding new contributors to the miRTop small RNA analysis tool, Galaxy user interface improvements with initial collaborative work with the JBrowse genome browser, creation of multiple new Galaxy training tutorials, and additions to the Common Workflow Language ecosystem for provenance and dependency management. We received overwhelmingly positive responses to the post-CoFest survey, highlighting the improved integration with the conference. Comments included:

*This is the most productive part for me of the conference. I'm so glad it was extended to be more days than it used to be. Moving it to the end of the conference was also a good thing.*
*Having physical proximity to experts and those who are involved in the code and project was excellent!*

## Feedback

With its commitment to openness, BOSC is always receptive to input from the community about what they want from the conference. To solicit feedback from those who didn’t attend GCCBOSC 2018, as well as those who did, the organizers put together an extensive questionnaire asking attendees what they thought of the meeting and asking non-attendees what they’d like to see in the future. 102 people filled it out, including 31 who didn’t attend GCCBOSC. Slightly more than half of the 71 respondents who did attend the meeting indicated that they attended primarily BOSC sessions, which matches the overall pattern of attendance (52% BOSC / 48% GCC).

**Figure 12.**
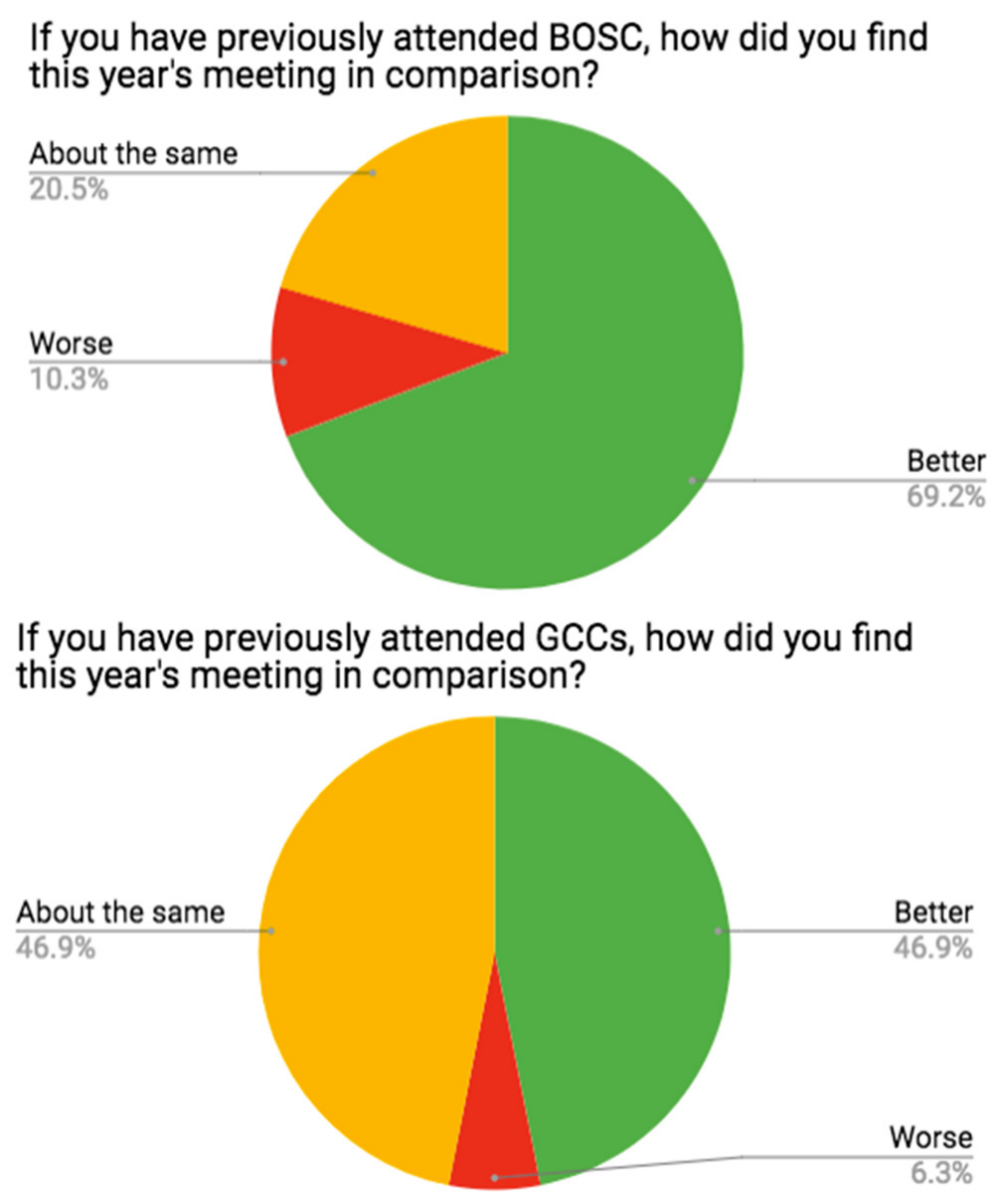
Responses to post- Galaxy Community Conference and Bioinformatics Open Source Conference (GCCBOSC) survey questions from those who had been to previous Galaxy Community Conferences (GCCs) or previous Bioinformatics Open Source Conferences (BOSCs).

There was wide agreement among meeting participants that the meeting was informative, productive and enjoyable. Most of the survey respondents who had previously attended a BOSC rated this year’s meeting as similar or better than past meetings (as did previous GCC attendees)--see Figure 12. The training workshops and extended CoFest were mentioned by participants as great features of the meeting. The main complaint was that the parallel GCC and BOSC sessions forced attendees to choose between them--an embarrassment of riches.

## What next?

As an experiment, GCCBOSC 2018 was a big success. Participants were overwhelmingly positive about the experience, and the conference attracted a somewhat different mix of attendees than in past years. However, we also concluded that there are some advantages to meeting with ISMB--for example, it attracts more students and postdocs, and the presence of other COSI tracks provides a wider range of scientific topics. Moreover, unlike the GCC 2018 venue, the venue already chosen for GCC 2019 has a number of drawbacks: we wouldn’t be able to run similarly-sized parallel sessions; registration prices wouldn’t be as affordable as in 2018; and the venue would not be able to accommodate the larger (160 people) and longer (four days) CollaborationFest that was one of the highlights of GCCBOSC 2018.

For these and other reasons, the BOSC organizing committee concluded that the best way to serve the broadest community of potential BOSC attendees will be to partner some years with International Society for Computational Biology (ISMB) and some with GCC. We therefore plan to hold BOSC 2019 in Basel as part of ISMB. We hope to partner with GCC in 2020 at a North American site to be determined, or in 2021 in Europe.

Wherever we end up meeting, we look forward to many more years of bringing together scientists, developers, users, data generators and more to discuss open science and open bioinformatics.

## Figures

Except where noted, all Galaxy Community Conference and Bioinformatics Open Source Conference (GCCBOSC) photographs in this report are from Bérénice Batut’s Flickr album, under a CC-BY-SA license. All identifiable subjects in the photos were contacted and they consented to have their photos used in this report.

